# Orally consumed cannabinoids: the effect of carrier oil on acute tissue distribution in male C57BL/6 mice

**DOI:** 10.1186/s42238-025-00298-4

**Published:** 2025-07-01

**Authors:** Cody A. C. Lust, Lyn M. Hillyer, Mitchell Pallister, Amanda J. Wright, Michael A. Rogers, Erin M. Rock, Cheryl L. Limebeer, Linda A. Parker, David W. L. Ma

**Affiliations:** 1 Department of Human Health and Nutritional Sciences, University of Guelph, Guelph, ON N1G2W1 Canada; 2 Department of Food Science, Guelph, Canada; 3 Department of Psychology and Collaborative Neuroscience Program, ON Guelph, N1G2W1 Canada

**Keywords:** Cannabinoids, CBD, THC, Tissue distribution, Omega-3, Sesame, Mice, Oral

## Abstract

**Background:**

Fundamental gaps in knowledge exist in understanding the tissue distribution of cannabinoids, cannabidiol (CBD) and tetrahydrocannabinol (THC), following oral ingestion. CBD and THC are lipid-soluble and oral bioavailability is increased when combined with long-chain fatty acid carrier oils prior to oral ingestion. Oils with eicosapentaenoic acid (EPA) and docosahexaenoic acid (DHA) confer positive health benefits and have yet to be examined as a carrier oil for oral cannabinoid delivery thus, examination is warranted.

**Methods:**

This study investigated the acute tissue distribution of cannabinoids in serum, adipose, brain, liver, heart, and muscle of male C57BL/6 mice at 1, 2, and 3 h (H) post oral ingestion. Mice were gavaged with CBD (5 mg/kg) and THC (1 mg/kg) combined with either sesame (SES), mixed EPA/DHA, or DHA enriched (DHA) oil as a carrier. With assistance of the Analytical Facility for Bioactive Molecules (Toronto, Canada), tissue concentration of cannabinoids was quantified using liquid chromatography with tandem mass spectrometry.

**Results:**

SES oil resulted in a significantly greater concentration of CBD and THC (*p* < 0.05) across all tissues and times compared to the n-3 polyunsaturated fatty acid (PUFA) oils. The ratio of EPA:DHA in the carrier oils modestly affected distribution of cannabinoids to tissues, notably, DHA oil resulted in a greater concentration of CBD in the brain. Heart tissue had the highest concentration of CBD at 1 and 2H post-oral gavage, and adipose tissue had the highest concentration at 3H which was consistent across all three carrier oils.

**Conclusions:**

This study profiled the greatest number of tissues to-date for the acute distribution of CBD and THC following oral consumption with a lipid carrier in mice which demonstrated a non-uniform distribution to tissues over time. SES oil proved to be far more effective as a carrier oil at delivering orally consumed cannabinoids to tissues compared to two different n-3 PUFA containing oils. Further developing our fundamental understanding of cannabinoid distribution across tissues following their consumption from foods and pharmaceuticals are necessary to establish specific pharmacokinetic profiles to aid oral dosing strategies and maximize the bioactive potential of cannabinoids.

## Introduction

Federal legalization of recreational cannabis in Canada and an expanding number of states in the United States of America has resulted in numerous cannabis-based products in the marketplace (Hammond et al. 2022). The increased availability corresponds to increased cannabis sales for recreational and therapeutic applications to reduce pain and inflammation (Lötsch et al. 2018; Rotermann 2019). There are > 100 unique cannabinoids isolated; however, the most comprehensibly studied and commercially available products contain the primary psychoactive Δ−9-tetrahydrocannabinol (THC) and the non-psychoactive cannabidiol (CBD) (Chayasirisobhon 2020; Millar et al. 2018). Following ingestion, both cannabinoids are predominantly metabolized in the liver, with THC being converted into the psychoactive metabolite 11-hydroxy-THC, which is further oxidized to produce the non-psychoactive metabolite 11-nor-9-carboxy-THC (Huestis 2007). The psychoactive effects of THC acts as a partial agonist of the cannabinoid receptor CB_1_, a G-protein coupled receptor (GPCR) in the endocannabinoid system (ECS), which is expressed throughout the central nervous system (Lötsch et al. 2018; Lucas et al. 2018). CBD differs from THC by acting as a negative allosteric modulator of CB_1_ and CB_2_ receptors; both are widely expressed centrally and peripherally (including the gastrointestinal tract), whereas CB2 receptors are predominantly expressed in the immune system and are found to be upregulated in the central nervous system during disease or injury (Chayasirisobhon 2020; Lucas et al. 2018; DiPatrizio 2016). Neuroprotective and anti-inflammatory effects of CBD are agonists of the serotonin GPCR 5-HT_1A_ and the vanilloid TRPV1 receptors in the central nervous system (Silvestro et al. 2020). As cannabinoids are likely to be taken up across all tissues, driving different effects, understanding the rate, concentration, and distribution is important (Huestis 2007).

Inhalation was traditionally the most common mode of consumption for cannabis, but recent trends show that consumption of consumable products, such as edibles and drinks, is rising (Tassone et al. 2023; Hall et al. 2023). The mode of consumption/delivery (inhalation, transdermal, oral) of cannabinoids alters their metabolization significantly, impacting both the kinetics and overall bioavailability, thereby exerting differential biological effects (Lucas et al. 2018; Hlozek et al. 2017). The transit time of digestion has a signifcant lag preceeding the onset of uptake of orally consumed cannibinoids and a delayed peak concentration (C_max_) of cannabinoids in serum and the brain; contrasted to inhalation, which presents a much shorter lag and more rapid increase in bioavailability (Huestis 2007). Cannabinoids undergo significant first-pass metabolism in the liver following oral consumption in water-based beverage products, limiting bioavailability to < 10%, while inhaled cannabinoids bypass the liver, resulting in greater bioavailability of up to 31% for CBD and 56% for THC (Millar et al. 2018; Huestis 2007). Both CBD and THC are liposoluble, and when combined with long-chain fatty acid carrier oils, including vegetable based cooking oils such as sesame (SES), oral bioavailability can increase upwards of threefold (Zgair et al. 2016; Feng et al. 2021). Ingestion of dietary lipids is a known strategy to promote the absorption of many lipophilic bioactive molecules (e.g., micronutrients, pharmaceuticals) through mechanisms which enhances the solubility of bioactive molecules with the products of lipid digestion, promoting absorption and distribution through the lymphatic system (Hashida et al. 2005). As carrier oils are digested, they must be emulsified, forming lipid droplets, where digestive hydrolytic enzymes reduce the particle size, forming chylomicrons (75–1200 nm) containing cannabinoids that enter the lymphatic system bypassing the liver, reducing first-pass metabolism and increasing bioavailability (Martins et al. 1996; Gugliucci 2023). Once in circulation, CBD and THC are capable of distributing to a large number of organs and tissues, such as the lungs, heart, adipose tissue, and liver, and across the blood–brain barrier (BBB) in both animals and humans (Chayasirisobhon 2020; Kreuz and Axelrod 1973; Lust et al. 2022; Ruiz et al. 2021). Studies predominantly focus on cannabinoid concentration in the blood (serum/plasma) and brain tissue with limited reference to other tissues where cannabinoids have plausible relevance to human health (Hlozek et al. 2017; Dumbraveanu et al. 2023).

Oils containing n-3 polyunsaturated fatty acids (PUFA), specifically eicosapentaenoic acid (EPA) and docosahexaenoic acid (DHA), confer positive health benefits and are composed of long-chain fatty acids which is similar to the composition of other common vegetable oils (Shahidi and Ambigaipalan 2018). Vast literature supports a role for n-3 PUFA which correlates with reduced inflammatory markers, lowered blood triglycerides, and improved endothelial function, resulting in a lowered rate of cardiovascular disease (CVD) (Innes JK 2020). DHA is enriched in brain phospholipids and is an integral component of neural function, development, and mediator of the ECS in the central nervous system (Dyall 2017; Sun et al. 2018). Commercial products containing mixtures of n-3 PUFA and cannabinoids are already sold with associated health claims. However, their metabolism and transport in the body when combined is unknown as n-3 PUFA containing oils have yet to be examined as a carrier oil in conjunction with orally administered cannabinoids. A better understanding of how carrier oils affect cannabinoid bioavailability and distribution across multiple tissues post-oral consumption is highly relevant to the therapeutic potential for cannabinoids in human disease as well as toxicity. This study examines the acute distribution of major cannabinoids and metabolites across six major tissues after 1, 2, and 3 h (H) following oral gavage. Two different ratios of n-3 PUFA oils are compared to SES oil as carriers of CBD (5 mg/kg body weight [BW]) and THC (1 mg/kg BW) to assess the feasibility of attaining human doses in male C57BL/6 mice.

## Material and Methods

### Animals

All animal use protocols (#4687) are approved by the Institutional Animal Care Committee at the University of Guelph, which the Canadian Council on Animal Care accredits, and a cannabis use permit allows for the handling, administration, and storage of cannabis (CANN 02 MA). Male C57BL/6 mice were obtained from Charles River Laboratories (St. Constant, QC, Canada). All mice, n = 6 at each time point and for each carrier oil, were fasted from food for 6 H before oral gavage with cannabinoids.

### Cannabinoids and Oil

Purified CBD (97.4% pure) and THC (98% pure) obtained from Toronto Research Chemicals (Toronto, ON, CAN) contained in ethanol (EtOH) were mixed in one of three carrier oils: Pure SES oil (control) (Sigma-Aldrich—St. Louis, MO, USA), life's Omega™ 60 (EPA/DHA; 21.5% EPA, 34.7% DHA), or, life's DHA™ (DHA enriched; 1.6% EPA, 43.7% DHA) (composition provided by DSM Nutritional Products, Columbia, MD, USA). Mixtures were prepared in small batches daily by combining pure cannabinoids contained in EtOH (< 1% v/v of mixture) directly with the carrier oil at the desired ratio and vortexed for ~ 5 min. The prepared mixture was administered via oral gavage to mice at 5 mg/kg BW for CBD and 1 mg/kg for THC. Compared to other studies using rodent models, the relatively low CBD and THC doses are chosen to reflect the upper range of physiologically feasible acute human CBD and THC intake (Gorelick et al. 2013).

### Tissue and Blood Collection

Mice were asphyxiated via CO_2_ at 1, 2, or 3H post-oral gavage, after which the brain, adipose (epididymal), liver, heart, muscle (quadriceps), and whole blood were immediately collected, flash-frozen in liquid nitrogen (excluding serum) and stored at −80 °C. The heart was flushed with phosphate-buffered saline (PBS) to expel blood remaining in the chambers before flash freezing. Collected whole blood was centrifuged at 13,000 × *g* for 5 min to separate phases, and the serum was pipetted into a clean 1.5 mL Eppendorf vial.

### Extraction and Quantification of Metabolites by LC–MS/MS

The method to extract and quantify CBD and THC from tissues is adapted and can be found in full from Kraemer, Broecker (Kraemer et al. 2019) and was performed at SPARC BioCentre, Hospital for Sick Children, Toronto, Canada. Frozen samples were homogenized and then weighed, 20 mg tissues and 50 µL for serum, and then suspended with 100 ng/ml of THC-D3 internal standard (Sigma-Aldrich [St. Louis, MO, USA]) in H_2_O. 1 M HCl followed by 9:1 (v/v) hexane/ethyl acetate were added, centrifuged, and the supernatant was removed and repeated combining the supernatants. Samples and standards were then evaporated under a gentle flow of nitrogen. Residues were reconstituted in 120 µL acetonitrile and centrifuged with the remaining supernatant transferred into glass inserts for liquid chromatography with tandem mass spectrometry (LC–MS/MS) analysis. An Agilent 1290 UPLC system (Agilent Technologies, Santa Clara, CA, USA) fitted with a Sciex Q-Trap 5500 mass spectrometer (MS) (AB Sciex, Framingham, MA, USA) was used in Electrospray Ionization mode, positive mode. The MS transitions and collision-induced dissociation for each compound were optimized with standard substances. Detections were performed using scheduled multiple reaction monitoring mode. The following ion source settings were used: ion source (turbo spray), curtain gas 40 psi, ion source gas 1 60 psi, ion source gas 2 40 psi, ion spray voltage 4500 V, source temperature 600 °C. Samples were separated using a Kinetex Biphenyl column (2.6 µm, 100 Å, 50 × 2.1 mm; Phenomenex, Torrence, CA). A gradient mobile phase of 5 min at a flow rate of 0.4 ml/min was used for the elution of cannabinoids with mobile phase A: 0.1% formic acid in water and mobile phase B: 0.1% formic acid in acetonitrile. The mobile phase gradient was: t = 0 min 50% B, t = 2.50 min 95% B, t = 2.55 min 50% B, and t = 4.50 min 50%. All LC–MS/MS grade solvents were purchased from Caledon Laboratories Ltd (Georgetown, ON). The standard curve range was: 0.01–200 ng (THC and CBD). Data was acquired and analyzed by Analyst v 1.6.2 (Sciex).

### Data Analysis

Statistical analysis was conducted using SAS OnDemand for Academics (SAS Institute Inc., Cary, NC, USA). Tissue values are expressed as ng/g, and serum values as ng/ml. Values outside of the highest/lowest point on the standard curve of the LC–MS/MS analysis were removed. Further, outliers based on the 1.5 interquartile range (IQR) method, values below the 25th percentile minus 1.5 times the IQR or above the 75th percentile plus 1.5 times the IQR, were removed (Alabrah 2023). After removing outliers, any cannabinoid and carrier oil group consisting of < 3 samples was not used in the statistical analysis and was identified as not calculated (n/c). A two-way analysis of variance (ANOVA) was conducted to determine the main effects (*p* < 0.05) of carrier oil SES, EPA/DHA, DHA and time (1, 2, and 3H) on cannabinoid concentrations in various tissues. If a significant (*p* < 0.05) interaction was not found, a one-way ANOVA was conducted for significant main effects, followed by Tukey's post-hoc analysis.

## Results

### Cannabidiol

There were main effects of carrier oil and time (*p* < 0.05) on CBD concentration across all six tissues (Table 1). Of the six tissues assessed, a statistically significant interaction effect (*p* < 0.05) was found for adipose, muscle, liver, and serum and not for the heart and brain. Two-way ANOVA analysis showed that SES carrier oil at 2H results in significantly (*p* < 0.05) greater CBD tissue concentrations than all other carrier oil-time combinations for liver, muscle, and serum. Adipose tissue has a statistically greater concentration (*p* < 0.05) of CBD delivered by SES at 3 H compared to all other carrier oil and time combinations. There was no significant interaction of carrier oil and time in heart and brain tissue, but individually carrier oil and time were significantly different (*p* < 0.05). Thus, further analysis by one-way ANOVA of carrier oil and time followed by Tukey post-hoc was conducted for the heart and brain (Table 1). At each timepoint for heart and brain tissue, CBD concentrations with SES oil carrier were significantly greater (*p* < 0.05) than both n-3 PUFA oils (results not displayed in Table 1), while there is no significant difference between the two n-3 PUFA carrier oils in any of the six tissues across all timepoints. On average, EPA/DHA oil resulted in a greater C_max_ of CBD when compared to DHA oil (Table 1), with a notable exception where the DHA oil led to greater C_max_ of CBD in the brain at 2H and 3H than EPA/DHA oil. Overall, SES oil was associated with higher postprandial CBD concentrations at all three timepoints across all six tissues. The greatest C_max_ of CBD is in heart tissue at 1 and 2H across all three oils (Table 1). A notable shift in C_max_ occurs at 3H, with adipose tissue having the greatest concentration of CBD for all carrier oils. The tissue with the lowest C_max_ of CBD was consistently the brain. Similar trends in the time to reach peak concentration (T_max_), 2H post-oral gavage in five tissues, excluding adipose tissue, with a T_max_ at 3H, were observed across all three carrier oils (Table 1).

**Table 1:** Mean concentration of CBD over time in multiple tissues following oral consumption with each carrier oil

Tissue	Carrier Oil	1H	2H	3H	*p*-value Interaction	*p*-value Carrier Oil	*p*-value Time
Adipose (ng/g)	SES	33.8 ± 12.2^bc^	118.3 ± 61.6^ab^	205.8 ± 120.7^a^	0.01*	< 0.01*	< 0.01*
	EPA/DHA	5.7 ± 2.7^c^	15.5 ± 9.6^c^	50.4 ± 34.0^bc^			
	DHA	6.2 ± 4.5^c^	20.6 ± 9.2^c^	29.0 ± 18.7^bc^			
Brain (ng/g)	SES	11.4 ± 4.7	30.3 ± 11.3	27.1 ± 7.4	0.32	< 0.01*	< 0.01*
	EPA/DHA	4.0 ± 1.1	8.3 ± 4.2	7.4 ± 2.5			
	DHA	2.5 ± 0.6	11.1 ± 11.8	18.4 ± 21.0			
Heart (ng/g)	SES	103.4 ± 25.6	231.8 ± 82.4	182.4 ± 111.0	0.07	< 0.01*	0.02*
	EPA/DHA	24.2 ± 7.8	45.5 ± 34.0	22.6 ± 8.6			
	DHA	15.8 ± 5.6	25.1 ± 19.4	17.8 ± 7.3			
Liver (ng/g)	SES	25.3 ± 10.2^b^	72.8 ± 36.6^a^	30.3 ± 18.7^b^	0.04*	< 0.01*	< 0.01*
	EPA/DHA	10.2 ± 5.8^b^	27.9 ± 21.3^b^	13.9 ± 4.5^b^			
	DHA	7.7 ± 3.8^b^	18.5 ± 5.6^b^	25.6 ± 9.0^b^			
Muscle (ng/g)	SES	19.6 ± 5.3^b^	51.8 ± 8.2^a^	59.5 ± 24.8^a^	< 0.01*	< 0.01*	0.01*
	EPA/DHA	21.2 ± 15.5^b^	14.3 ± 4.9^b^	11.5 ± 3.7^b^			
	DHA	6.0 ± 2.5^b^	15.3 ± 11.2^b^	10.8 ± 4.3^b^			
Serum (ng/ml)	SES	26.0 ± 13.6^b^	91.6 ± 42.2^a^	44.6 ± 31.3^b^	0.01*	< 0.01*	< 0.01*
	EPA/DHA	6.7 ± 4.3^b^	19.3 ± 19.1^b^	16.1 ± 17.4^b^			
	DHA	6.2 ± 4.7^b^	9.1 ± 6.5^b^	5.0 ± 2.7^b^			

Tissues concentrations of CBD are expressed as ng/g ± SD. Serum concentrations are expressed as ng/ml ± SD. *Two-way ANOVA assessed the main effects of carrier oil and time within each tissue (*p* < 0.05). ^abc^When there was a significant interaction, differences between all oil*time combinations within a single tissue were determined by Tukey post hoc (p < 0.05). Values with different superscript letters are significantly different (*p* < 0.05). There was no significant interaction observed in brain and heart tissues, thus one-way ANOVA for significant main effects followed by Tukey was conducted. At each timepoint for heart and brain tissue, SES was significantly greater (p < 0.05) than both n-3 PUFA oils (not displayed). *n* = 4–6 for all combinations of carrier oil and time

### Tetrahydrocannabinol

The main effect of carrier oil(s) is significant (*p* < 0.05) for THC concentrations across all 6 tissues (Table 2), while the main effect of time is significant in 3 tissues: adipose, brain, and liver. The only significant interaction (*p* < 0.05) is found in the liver. Two-way ANOVA analysis shows liver THC concentration is significantly greater (*p* < 0.05) when combined with SES oil than n-3 PUFA oils at all time intervals (Table 2). The only significant difference between EPA/DHA and DHA carrier oils is at 3H in the liver, where tissue concentration of THC is significantly greater (*p* < 0.05) for DHA than EPA/DHA oil. Further analysis by one-way ANOVA with Tukey post-hoc for the remaining five tissues show no significant interaction (Table 2). Of the calculatable time points (i.e., n/c time points (Table 2) were excluded), significantly greater (*p* < 0.05) tissue concentrations of THC are observed for SES carrier oil across all times and tissues compared to EPA/DHA and DHA carrier oils (results not shown). SES oil is significantly more effective at increasing THC concentrations post-oral consumption across all times and in all tissues, mirroring the CBD results (Table 1). THC C_max_ is more variable than CBD (Table 1), and T_max_ most commonly occurs 3H post-oral gavage.

**Table 2:** Mean concentration of THC over time in multiple tissues following oral consumption with each carrier oil

Tissue	Carrier Oil	1H	2H	3H	*p*-value Interaction	*p*-value Carrier Oil	*p*-value Time
Adipose (ng/g)	SES	7.6 ± 5.4	15.8 ± 5.0	27.8 ± 9.4	0.08	<.01*	<.01*
	EPA/DHA	1.7 ± 0.7	4.5 ± 2.7	4.5 ± 2.2			
	DHA	1.9 ± 1.3	3.7 ± 2.3	19.0 ± 16.7			
Brain (ng/g)	SES	3.4 ± 0.7	5.6 ± 1.6	7.9 ± 1.8	n/c	<.01*	<.01*
	EPA/DHA	n/c	1.5 ± 0.4	n/c			
	DHA	n/c	n/c	1.9 ± 0.5			
Heart (ng/g)	SES	12.1 ± 4.5	14.7 ± 6.2	22.7 ± 11.8	0.40	<.01*	0.06
	EPA/DHA	5.4 ± 2.2	3.3 ± 0.5	5.2 ± 1.0			
	DHA	1.2 ± 0.3	2.4 ± 1.1	3.4 ± 0.2			
Liver (ng/g)	SES	25.3 ± 4.2^a^	31.1 ± 3.7^a^	27.7 ± 1.6^a^	<.01*	<.01*	<.01*
	EPA/DHA	6.0 ± 2.9^b^	7.4 ± 3.2^b^	7.6 ± 2.9^b^			
	DHA	12.1 ± 7.4^b^	7.3 ± 2.4^b^	32.0 ± 16.3^a^			
Muscle (ng/g)	SES	5.0 ± 2.1	4.9 ± 2.5	5.8 ± 1.0	0.30	<.01*	0.56
	EPA/DHA	n/c	2.3 ± 1.0	1.9 ± 0.4			
	DHA	2.0 ± 0.3	4.1 ± 3.3	1.7 ± 0.6			
Serum (ng/ml)	SES	5.8 ± 2.4	5.3 ± 1.1	4.9 ± 1.2	n/c	0.02*	0.89
	EPA/DHA	n/c	3.4 ± 2.7	n/c			
	DHA	n/c	n/c	1.8 ± 0.7			

Tissues concentrations of THC are expressed as ng/g ± SD. Serum concentrations are expressed as ng/ml ± SD. *Two-way ANOVA assessed the main effects of carrier oil and time within each tissue (*p* < 0.05). ^abc^When there was a significant interaction, differences between all oil*time combinations within a single tissue were determined by Tukey post hoc (*p* < 0.05). Values with different superscript letters are significantly different (*p* < 0.05). *n* = 3–6 for all combinations of carrier oil and time. n/c = unable to be calculated

## Discussion

This study investigated CBD and THC tissue distribution at 1, 2, and 3H and the efficacy of EPA/DHA and DHA oil compared to SES carrier oils on the acute incorporation of orally consumed cannabinoids in the tissues of mice. SES oil significantly increased concentrations (*p* < 0.05) of CBD and THC in all six tissues across most time points compared to either n-3 PUFA carrier oils (Tables 1 and 2). When compared, EPA/DHA oil performed marginally better than DHA oil at increasing CBD and THC tissue concentrations; however, DHA oil notably resulted in higher brain cannabinoid concentrations. THC tissue concentration was highly variable and sometimes below the standard curve range of the analysis method; thus, CBD is primarily discussed. Overall, SES oil is far more effective at increasing tissue concentrations of cannabinoids following oral consumption. However, differential trends of incorporation between the two different ratios of EPA and DHA found in the n-3 PUFA oils demonstrate potential for future modifications to improve or tailor delivery to tissues.

Based on current knowledge, this study is the first to assess n-3 PUFA carrier oil to increase the bioavailability of orally consumed CBD and THC. Several other vegetable oils consisting of long and medium-chain triglycerides have been assessed in rodent models with varying success at increasing the bioavailability of cannabinoids, the most effective being SES and olive oil (Zgair et al. 2016; Izgelov et al. 2020; Feng et al. 2022). SES oil was chosen as the control to compare against the two n-3 PUFA oils as SES oil is established in the literature as an effective carrier oil and is used in the only drug containing CBD (Epidiolex®) approved by the US Food and Drug Administration and European Medicines Agency (Zgair et al. 2016; Lattanzi et al. 2021; Silmore et al. 2021). The results presented here conform with previous research, which shows SES oil is a superior carrier oil and promotes postprandial incorporation of for CBD and THC in the liver, serum, and brain. Additionally, this effect is observed in other tissues, including heart, muscle, and adipose, and the degree and speed of uptake differ between tissues, making it relevant when studying the impact of CBD and THC on a tissue-specific basis, related to mechanisms of action and managing potential risk with oral ingestion of cannabinoids (Froude et al. 2024).

As n-3 PUFA oil contains the long-chain PUFA, EPA and DHA, we hypothesized that the chylomicrons formed during digestion would allow the incorporated cannabinoids to enter directly into the lymphatic system, reducing the first-pass metabolism in the liver, resulting in tissue concentrations that were in a similar range to that of SES oil (Feng et al. 2021). Vegetable oils vary in fatty acid composition and distribution, leading to distinct physical properties (i.e. density of the chylomicrons formed) that alter intestinal lymphatic transport of co-administered cannabinoids post-oral consumption (Feng et al. 2022). SES oil is typically composed of > 80% unsaturated fatty acids, ~ 47% of n-6 PUFA are linoleic acid (LA; 18:2n6) and monounsaturated fatty acid (MUFA) are ~ 37% oleic acid (OA; 18:1n9) (Wei P et al. 2022; Oboulbiga et al. 2023). The EPA/DHA and DHA oil are > 60% n-3 PUFA and < 10% MUFA, a notable difference from SES oil. Previous research in rats found corn oil, consisting of OA and LA, and cod liver oil, of EPA and DHA, have similar quantities of triglycerides recovered from intestinal lymph 6H post-ingestion, indicating similar absorption profiles despite differences in fatty acid profiles. (Degrace et al. 1996). Additionally the largest chylomicrons formed are with corn oil, while cod liver oil results in smaller, more numerous chylomicrons (Degrace et al. 1996). Also, rats fed a diet rich in olive oil, with high levels of OA, had twice the lymphatic transport rate of FA at 8 H compared to those fed a high n-3 PUFA diet (Porsgaard and Hoy 2000). Research conducted *in-vitro* with Caco-2 cells found that EPA and DHA limit chylomicron assembly and secretion, whereas OA promotes chylomicron formation (Wang et al. 2014; Williams et al. 2004). Similar results in humans found orally consumed EPA and DHA decreased chylomicron size while accelerating clearance and reducing intestinal lymphatic transport of n-3 PUFA (Park and Harris 2003). Therefore, oils high in n-3 PUFAs and low in MUFAs limited lymphatic transport of co-administered cannabinoids, accounting for the lower tissue concentrations observed for EPA/DHA and DHA in the current study compared to that of SES oil.

The lower experimental doses are chosen to reflect quantities toleratable to acute human intake, which still elicit physiological effects reported in rodents (Dumbraveanu et al. 2023; Gorelick et al. 2013; Rock et al. 2020; Moore and Weerts 2022). A greater dose of CBD over THC was chosen because CBD is non-psychoactive, and previous research shows it is tolerated at higher doses in humans, with no significant side effects in adults orally consuming acute doses of 600–1500 mg of CBD (Bergamaschi et al. 2011). If an average adult male weighing 85 kg were to consume the dose used in our study, 5 mg/kg of CBD, that would equate to an acute dose of 425 mg, well within tolerable quantities. Previous studies using rodents provide an oral dose between 20–100 mg/kg, an unrealistic to achieve in humans (Yau et al. 2023). When considering chronic supplementation of CBD, research recommends more conservative doses of 30–160 mg/day CBD to be considered safe in healthy adults (Henderson et al. 2023). Additionally, in countries where recreational cannabis products are sold, the 425 mg quantity used in this study far exceeds the typical quantity of CBD sold in consumable products requiring consumers to purchase numerous items to achieve the same dosage (Tassone et al. 2023). Future studies must consider the upper dose limit of cannabinoids, route of administration, and study duration (acute vs. chronic) while maintaining the safety and applicability of results to healthy adults.

For the first time, the tissue distribution of major cannabinoids in six different tissues following oral consumption of CBD and THC with a lipid carrier shows a wide distribution of CBD and THC after 1H post-oral gavage (Tables 1 and 2). With CB_1_ and CB_2_ receptors being widely distributed throughout many tissues, it is important to consider the whole-body perspective, as many targets exist for cannabinoids to interact with and influence biological function and outcomes (Kilaru and Chapman 2020). Irrespective of carrier oil, T_max_ is most common at 2H post-oral gavage, which aligns with previous pharmacokinetic studies reporting T_max_ between 90–120 min (Millar et al. 2018; Lucas et al. 2018). Comparison between studies is challenging because of time intervals, lipid carrier, digestive conditions of each species, and cannabinoid dose each influence the outcome (Millar et al. 2018). A wide cannabinoid dose range from 1 mg/kg to > 100 mg/kg, is used across experiments, which dramatically impacts C_max_ in tissues as oral administration of cannabinoids is dose-dependent and variable (Millar et al. 2018; Huestis 2007; Yau et al. 2023). Studies of a similar CBD oral dosage, 15 mg/kg of CBD mixed with SES oil, present a T_max_ at 4H and a plasma/serum concentrations, C_max_ of 135.94 ± 38.15 ng/ml in rats (Fu et al. 2022). In rats, a 12 mg/kg CBD dose in SES oil reports a T_max_ at 3H post-oral gavage and a C_max_ of 164 ± 142 ng/ml (Brookes et al. 2023). The pharmacokinetic profile of an orally-gavaged 10 mg/kg CBD dose administered to rats in two separate studies both found T_max_ at 2H post-oral gavage and a C_max_ of 96.5 ± 17.3 and ~ 275 ng/ml, respectively (Hlozek et al. 2017; Schwotzer et al. 2023). Orally-consumed CBD with SES carrier oil in our study resulted in a T_max_ at 2H and C_max_ of 91.6 ± 42.4 ng/ml in serum, while the serum C_max_ of CBD with n-3 PUFA carrier oils was 9.1 ± 6.5 ng/ml for DHA and 19.3 ± 19.1 ng/ml for EPA/DHA, well below the reported values mentioned above.

The central nervous system contains the highest concentration of cannabinoid receptors and has other receptors that cannabinoids, notably CBD, interact with (e.g., peroxisome proliferator-activated receptor gamma (PPARγ), transient receptor potential vanilloid 1 (TRPV1), and the serotonin 1 A receptor (5-HT1A), making increased delivery to the brain a research priority (Yau et al. 2023; Tambe et al. 2023). Along with serum, the distribution of orally consumed cannabinoids is commonly reported in brain tissue exhibiting dose-dependent responses in humans and animals (Millar et al. 2018). The C_max_ of CBD in brain tissue is reported as 126 ± 9.5 and ~ 225 ng/g and a T_max_ at 2H (Hlozek et al. 2017; Schwotzer et al. 2023). Cannabinoids used to treat pain are studied in mice at concentrations ranging from 1 to 20 mg/kg CBD in SES oil; the 20 mg/kg dose C_max_ is ~ 200 ng/g, while a 1 mg/kg dose C_max_ is ~ 3 ng/g in brain tissue, and a T_max_ of 4H and 2H, respectively (Dumbraveanu et al. 2023). Similar to our study, a 5 mg/kg oral CBD dose in SES oil has a C_max_ of 30.3 ± 11.3 ng/g in brain tissue with a T_max_ at 2H. SES oil has a significantly greater C_max_ in brain tissue at all times compared to the n-3 PUFA oils (Table 1). CBD C_max_ is lowest in the brain across time points compared to the other tissues, which may be due to the selectivity of the BBB compared to other tissues (Calapai et al. 2020). A greater C_max_ in heart and adipose tissues (Tables 1 and 2) implies cannabinoids are distributed to other tissues before the brain following digestion, reducing the concentration of accessible cannabinoids to the brain.

The non-specific tissue distribution following ingestion limits oral cannabinoid delivery; thus, strategies to increase bioavailability and enhance targeted delivery, including lipid-based nanoparticles and self-emulsifying drug delivery systems, are needed (Reddy et al. 2023). We hypothesized that due to the high concentration of DHA in brain phospholipids (Lacombe et al. 2018), oral CBD combined with a DHA oil compared to an equal ratio of EPA/DHA oil results in a greater C_max_ in brain tissue, which is confirmed at 2H and 3H (Table 1). The carrier oil used has found to alter cannabinoid delivery to specific brain regions where SES oil more effectively increases CBD in the hippocampus, while lipid-free formulations result in more CBD in the cerebellum (Brookes et al. 2023). Improving the bioavailability and selectivity of oral formulations will yield more targeted tissue distribution of cannabinoids and tailored health outcomes.

The tissue distribution of orally consumed cannabinoids is limited despite the increase in orally ingestible cannabis products on the market (Hammond et al. 2022). Heart tissue has the greatest CBD concentration for all carrier oils at 1H post-oral gavage (Table 1) (SES: 103.4 ± 25.6 ng/g; EPA/DHA: 24.2 ± 7.8 ng/g; DHA: 15.8 ± 5.6 ng/g). Cannabinoids are distributed into organs and tissues that are well vascularized, and since all blood must flow through the heart, the elevated concentration is not unexpected (Chayasirisobhon 2020; Kreuz and Axelrod 1973; Ruiz et al. 2021). CBD elicits many beneficial effects on heart health, such as lowering CVD risk via stimulating anti-inflammatory pathways of CB_2_ and TRPV1 receptors (Kicman A 2020; Fulmer and Thewke 2018). Dosages used in previous works report CBD concentration of adipose, liver, and muscle tissue upwards of 40 × greater than this study, making direct comparisons difficult due to the dose-dependent nature of orally consumed cannabinoids. At 2H post-oral gavage, heart tissue has the highest CBD C_max_ (SES: 231.8 ± 82.4 ng/g; EPA/DHA: 45.5 ± 34.0 ng/g; DHA: 25.1 ± 19.4 ng/g). At 3H, a notable shift shows an increased concentration of CBD in adipose tissue overtaking heart tissue concentration (SES: 205.8 ± 120.7 ng/g; EPA/DHA: 50.4 ± 34.0 ng/g; DHA: 29.0 ± 18.7 ng/g). Adipose tissue effectively stores cannabinoids long-term, with previous studies reporting cannabinoid concentrations in adipose days and even weeks post-ingestion (Kreuz and Axelrod 1973; Gunasekaran et al. 2009). Child and Tallon (Child RB 2022) found that at 28 days post-oral gavage of CBD given to rats significantly increased concentrations in adipose tissue compared to skeletal muscle (gastrocnemius) and the liver, supporting findings herein where adipose tissues reached their peak concentration at 3H. Although liver tissue has the third highest concentration of CBD after heart and adipose tissue, the C_max_ of THC is consistently the highest in the liver (Table 2). While CBD and THC share similar structural characteristics and metabolism (Huestis 2007), a greater metabolic rate in the liver for THC than CBD, coupled with more CBD excreted in feces, may account for the differences observed herein (Rao Q et al. 2022). Future studies examining the distribution and concentration of orally consumed cannabinoids in the liver and other relevant tissues are necessary to develop specific pharmacokinetic profiles that improve oral dosing.

### Limitations

High tissue concentration variability following oral consumption is previously reported and also found herein (Tables 1 and 2), especially in mice fed 1 mg/kg THC (Millar et al. 2018). Despite testing a physiologically feasible dose, the low THC dose led to some tissues not being quantifiable based on the calibration curve by LC–MS/MS. Phospholipids were not removed during tissue sample preparation which can affect detector response when assessed by LC–MS/MS. The use of individual labelled internal standards in future studies is also recommended to unambiguously identify and quantify metabolites. Although the mice were fasted, the lower cannabinoid dose, digestion variability, and quantification method must be considered in future studies. Other factors which may influence the digestion and absorption of cannabinoids were outside of the scope of this study, namely that the fatty acid composition of the carrier oil may alter their solubilization in the digestate, as shown in some in vitro digestion experiments (Feng et al. 2022). One limitation of this study is using only male C57BL/6 mice, as sex differences alter cannabinoid accumulation (Moore and Weerts 2022; Sallam et al. 2023). It is recommended that future dose–response studies quantify sex separately. In brain tissues, previous literature shows uneven cannabinoid accumulation in specific regions depending on the carrier oil and time course assessed (Brookes et al. 2023), rationalizing more specific examination of cannabinoid distribution within various tissue types.

## Conclusion

The effect of oral consumption of cannabinoids on acute tissue distribution is largely unexplored, and a greater understanding will enhance the bioactive potential of cannabinoids for human health. This study is the first to (a) describe the tissue distribution of orally consumed cannabinoids in multiple tissues and (b) assess the efficacy of n-3 PUFA oils as a carrier oil to increase bioavailability. The results illustrate a non-uniform acute distribution of cannabinoids across tissues after oral consumption, commonly reaching C_max_ at 2H. Despite serum concentrations of cannabinoids commonly being used as a proxy, it ineffectively translates to tissue distribution and overall bioactive potential. Additionally, n-3 PUFA delivers orally consumed cannabinoids to multiple tissues, while SES oil is a more effective carrier. Moreover, despite n-3 PUFA oils showing limited efficacy as a carrier oil, co-consumption with cannabinoids may still provide synergistic health benefits that warrants further exploration. Further efforts should examine sex differences to continue improving the bioavailability of orally consumed cannabinoids for recreational and therapeutic benefits. Future investigations into cannabinoid distribution should include more diverse tissues relevant to human health.

## Data Availability

Data pertaining to the results of this manuscript are available upon request of the corresponding author.

## Abbreviations

ANOVA: Analysis of variance
BBB: Blood-brain barrier
BW: Body weight
CBD: Cannabidiol
CVD: Cardiovascular disease
C_max_: Peak concentration
DHA: Docosahexaenoic acid
EPA: Eicosapentaenoic acid
ECS: Endocannabinoid system
GPCR: G-protein coupled receptor
H: Hours
IQR: Interquartile range
LA: Linoleic acid
LC–MS/MS: Liquid chromatography with tandem mass spectrometry
MS: Mass spectrometer
MUFA: Monounsaturated fatty acid
OA: Oleic acid
PBS: Phosphate-buffered saline
PPARγ: Proliferator-activated receptor gamma
PUFA: Polyunsaturated fatty acids
SES: Sesame
THC: Δ-9-Tetrahydrocannabinol
T_max_: Time to reach peak concentration
TRPV1: Transient receptor potential vanilloid 1
5-HT1A: Serotonin 1A receptor
